# First description of the complete mitochondrial genomes of the species *Amblyomma humerale* and *Amblyomma geayi* (Acari: Ixodidae), Amazon, Pará, Brazil

**DOI:** 10.1007/s10493-026-01151-w

**Published:** 2026-06-24

**Authors:** Lucas Henrique da Silva e Silva, Fábio Silva da Silva, Sandro Patroca da Silva, Paulo Henrique Gomes de Castro, Anita Roberta Stone, Leonardo Henrique Almeida Hernández, Daniel Damous Dias, Sâmia Luzia Sena da Silva, Lucia Aline Moura Reis, Hanna Carolina Farias Reis, Liliane Leal das Chagas, Tania Cristina Alves da Silveira da Cunha, Flávio Rodrigues da Costa, Bruno Tardelli Diniz Nunes, Bruna Laís Sena do Nascimento, Roberto Carlos Feitosa Brandão, Durval Bertram Rodrigues Vieira, Lívia Caricio Martins, Ana Cecília Ribeiro Cruz, Joaquim Pinto Nunes Neto

**Affiliations:** 1 Post-graduate program in Parasitary Biology in the Amazon, Center of Biological and Health Sciences, State of Pará University, Belém, 66095-662 Brazil; 2 Department of Arbovirology and Hemorrhagic Fevers, Evandro Chagas Institute - IEC/SVS/MS, Ananindeua, 67030-000 Brazil; 3 National Primate Center – CENP, Ecology and Environment Section (SEEMA), Ananindeua, 67030-000 Brazil; 4 California Lutheran University, 60 West Olsen Road, Thousand Oaks, USA; 5 Federal Rural University of the Amazon, Parauapebas, 68515-000 PA Brazil

**Keywords:** Arbovirus. Entomology. Ixodida. Mitogenome. Tick-Borne

## Abstract

The genus *Amblyomma* koch, 1844 contains approximately 138 species, which are mainly distributed in the Afrotropical, Oriental, Neotropical and Australasian zoogeographic regions. This is an important group of arthropod vectors responsible for transmitting a wide range of etiological agents that cause infections and diseases in humans and animals, and is therefore of great interest to public and veterinary health.This study describes and characterizes, for the first time, the complete mitochondrial genomes of the species *Amblyomma humerale* and *Amblyomma geayi*. The obtained sequences presented a circular molecule structured in 37 functional subunits with 13 Protein-Coding Genes (PCGs), 22 transfer RNAs (tRNAs), two ribosomal RNA (rRNAs), two replication control regions, and two regions called “Tick-box”. The phylogeny reconstructed using the maximum likelihood method, based on the 13 concatenated PCGs, produced a monophyletic group for the genus *Amblyomma*, including the investigated sequences. *A. humerale* and *A. geayi* were grouped into the clades formed by the subgenera *Walkeriana* and *Anastosiella*, respectively. By integrating complete sequences, the approach adopted expands the number of comparable characters, reduces biases associated with the use of isolated mitochondrial markers, and contributes to better statistical support for the recovered nodes, resulting in more stable and interpretable phylogenetic topologies. The data and information generated in this study can contribute to future taxonomic and evolutionary research on the genus *Amblyomma*.

**Supplementary Information:**

The online version contains supplementary material available at 10.1007/s10493-026-01151-w.

## Introduction

Ticks (Arachnida: Ixodida) are specialized, exclusively hematophagous ectoparasites that feed on the blood of various vertebrate species (Dantas-Torres 2008; Brites-Neto et al. 2015; Orlova et al. 2023). They are a group of arthropod vectors of various pathogenic agents such as bacteria, protozoa, and viruses. These agents cause a wide range of infections and diseases in humans and animals, and are therefore of great interest to public and veterinary health (Springer et al. 2021; Schnittger et al. 2022; Silva et al. 2025a). The genus *Amblyomma* koch, 1844 contains approximately 138 species, which are mainly distributed in the Afrotropical, Oriental, Neotropical and Australasian zoogeographic regions (Guglielmone and Robbins 2018). Some species that are part of the genus *Amblyomma* receive special attention due to their involvement in the transmission of pathogens and the presence of cryptic species, which form important complexes, such as the species of the *Amblyomma cajennense* complex (Jerdy et al. 2023; Silva et al. 2025a).

The specie *Amblyomma geayi* Neumann, 1899, a parasitic tick of wild animals, is frequently associated with sloths (*Bradypus torquatus* and *Bradypus variegatus*) and primates (*Saimiri collinsi*) (Souza et al. 2016; Lima et al. 2023; Moreira et al. 2025). In addition, there are records in the literature of *A. geayi* nymphs parasitizing humans (Guglielmone et al. 2014; Guglielmone and Robbins 2018). Recently, the strain of *Rickettsia parkeri* from the Atlantic Forest was described in *A. geayi* collected from *Bradypus tridactylus* (Manaus, Brazil) (Moreira et al. 2025). However, little is known about the role of this species in the transmission of the etiological agent.

*Amblyomma humerale* Koch, 1844, is considered endemic to South America, with records in Brazil (Labruna et al. 2004; Lima et al. 2018; Machado et al. 2021), Bolivia (Robbins et al. 2003), Peru (Rojas-Jaimes et al. 2022), French Guiana (Koual et al. 2024), Suriname (Keirans 1984), Trinidad and Tobago (Robbins et al. 2003), Venezuela (Guerrero 1996) and Colombia (Benavides-Montaño et al. 2022). Records of *A. humerale* hosts include reptiles (Labruna et al. 2002; Machado et al. 2021), marsupials (Prati et al. 2023), rodents (Fuverki et al. 2021), and birds (Guglielmone et al. 2021). Although there are reports of *A. humerale* parasitizing humans, parasitism is considered rare (Guglielmone et al. 2021; Ferreira et al. 2025). Furthermore, etiological agents of the genera *Rickettsia* (Labruna et al. 2004) and Leishmania (Rojas-Jaimes et al. 2022) have been detected in *A. humerale* specimens. Although important infectious agents have been detected in this species, there is still no evidence to prove its vector competence (Labruna et al. 2004; Prati et al. 2023; Tojal et al. 2025).

The classic identification of ticks is based on morphological characteristics observed using microscopes (Brahma et al. 2014). However, this method has limitations in detecting immature or damaged specimens, as well as cryptic species (McCann et al. 2019; Monakale et al. 2024). On the other hand, modern approaches use mitochondrial DNA (mtDNA) molecular markers, enabling more precise taxonomy and more detailed phylogenetic analyses (Nava et al. 2015). The construction of phylogenetic relationships within the genus *Amblyomma* has been primarily based on rRNAs sequences (*12 S rRNA* and *16 S rRNA*), cytochrome c oxidase subunit I (*COXI*), widely used as a barcode on animals, and the cytochrome oxidase B gene (Nava et al. 2008; Lado et al., 2016, 2018; Ossa-López et al. 2022). Despite the wide availability of genetic information for several species of the genus *Amblyomma*, studies in this area have been limited, such that the complete mitochondrial genomes of some species have not yet been described in public databases. This limits the reconstruction of the genus’s evolutionary history. Therefore, the present study aims to describe and characterize the complete mitochondrial genome of two tick species of the genus *Amblyomma*.

## Methodology

### Obtaining tick samples

**Fig. 1:**
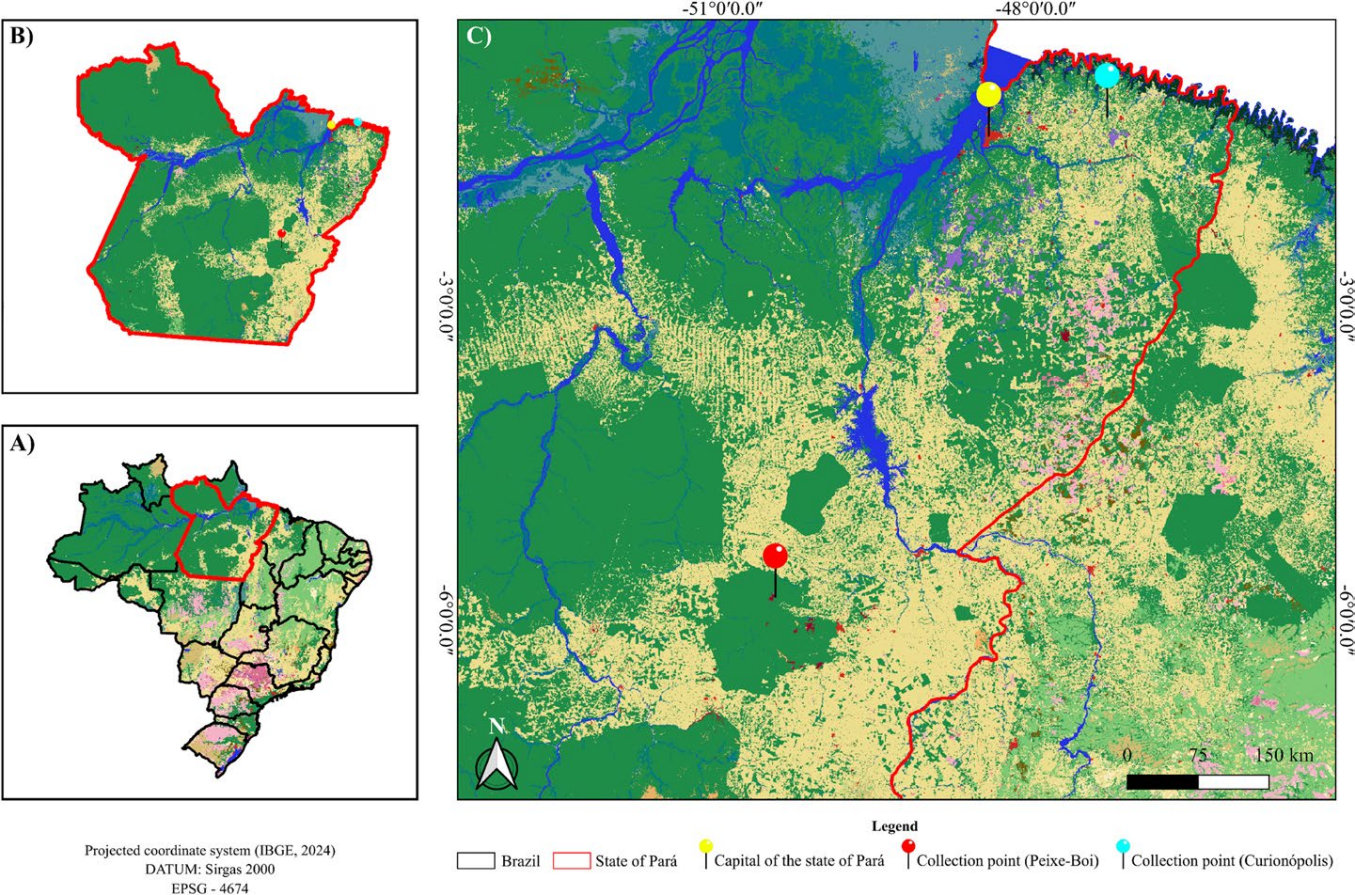
Location of the study area. **A**) Brazil; **B**) State of Pará; **C**) Indication of the collection points. The figure was created using QGIS v.3.16.16 (https://qgis.org/en/site/, accessed on May 13, 2026) with cartographic data from the Brazilian Institute of Geography and Statistics (IBGE, 2025) (https://www.ibge.gov.br/, accessed on May 13, 2026)

The tick samples are donations originating from two research projects. The first, entitled “Signals of genetic quality and mate choice” (ICMBio/SISBIO no. 82685), consists of a long-term monitoring study of a group of squirrel monkeys (*S. collinsi*), focusing on the behavioral ecology of *S. collinsi* (Stone et al. 2020). The *A. geayi* specimen (AR899865), donated by Ecology and Environment Section (SEEMA) from National Primate Center (CENP), was collected on January 11, 2024, from an adult female *S. collinsi* belonging to a group of squirrel monkeys, located in a forested area in the municipality of Peixe-Boi, Pará, Brazil. The second project is carried out by the Section of Arbovirology and Hemorrhagic Fevers (SEARB) of the Evandro Chagas Institute (IEC). The *A. humerale* sample (AR899867), donated by the project “Assessment of environmental changes and their relationship with the nosological profile in areas influenced by Vale enterprises – Salobo III Project, Serra Norte and Serra Leste,” was collected on November 17, 2022 (SEI/ICMBio no. 02122.001410/2019−35, ICMBio/SISBIO no. 82249−1) from an adult bird of the species *Saltator maximus*, captured in an area of secondary forest in the municipality of Curionópolis, Pará, Brazil (Fig. 1).

### Morphological and molecular identification of ticks

Morphological taxonomic identification was performed in the medical entomology laboratory of the Arbovirology and Hemorrhagic Fevers Section of the Evandro Chagas Institute. Specimens were identified by observing morphological characteristics using a stereomicroscope on a refrigerated table (−28 °C), using the dichotomous keys proposed by Martins et al. (2010) and Martins et al. (2013). Subsequently, the specimens were photographed and stored in a−80 °C freezer for preservation.

Molecular taxonomic identification was performed using the 5’ terminal region of the *COXI* gene (DNA barcode region). For this, sequences related to the barcode region of the genus *Amblyomma* were searched in the GenBank (NCBI) database. The sequences were manually evaluated with the Geneious software (v.11.1.5), and ten sequences phylogenetically close to the investigated sequences were used.

### Molecular Biology

#### Total RNA Extraction

A single specimen of each tick nymph species, *A. geayi* and *A. humerale*, was reserved and macerated (TissueLyser II) with 500 µL of Dulbecco’s phosphate-buffered saline solution (PBS), with 2% penicillin and streptomycin, 1% amphotericin B, 5% fetal bovine serum, and a sterile 3 mm diameter tungsten bead. The material was then centrifuged at 12,000 g at 4 °C for 10 min, and 140 µL of the supernatant was collected and purified using the QIAamp^®^ Viral RNA Mini Kit. After purification, the RNA was quantified using the Qubit RNA HS Assay Kit on the Qubit 4.0 instrument, following the manufacturer’s recommendations.

#### Double-stranded cDNA synthesis and genomic library preparation

The cDNA preparation from RNA was performed by synthesizing the first and second cDNA strands using the SuperScript™ IV VILO™ Second Strand cDNA Synthesis Kit. cDNA purification was performed using the PureLink^®^ PCR Purification Kit. All steps followed the recommendations of the respective kit manufacturers. The synthesized cDNA was quantified using the Assay DNA HS Kit on a Qubit 4.0 instrument. The genomic library was prepared following the guidelines of the Nextera XT DNA Kit and sequenced using the NextSeq 500 platform (Illumina, Inc.) with the NextSeq 500/550 High Output Kit v2.5 Kit (300 Cycles), employing paired-end sequencing as per the manufacturer’s recommendations.

### Bioinformatics

#### Data processing and characterization of mtDNA

The sequencing data obtained were initially subjected to a quality assessment using Fastp v.0.23.4 (Chen et al. 2018), conFigure d to remove adapter sequences and reads with a Phred base quality < 20 and a length less than 50 nt. Subsequently, the sequences were assembled de novo using MEGAHIT v.1.2.9 (Li et al. 2015) in its default execution configuration (k-mers of 21, 29, 39, 59, 79, 99, 119 and 141 nt). The mitochondrial contigs of the investigated species were identified using DIAMOND v.2.1.9.163 (Buchfink et al. 2021) (Blastx mode, e-value of 10⁻⁵) and manually inspected using Geneious v.11.1.5 (Kearse et al. 2012). Subsequently, they were annotated using the online tool MITOchondrial genome annotation Server (MITOS) (available at https://usegalaxy.eu/login/start) (Bernt et al. 2013) and circularized based on the identification of overlaps between the ends using Blastn v.2.16.0 (Camacho et al. 2009).

The circular structural representation of the obtained mitochondrial sequences was generated using CGview (Stothard et al. 2018), with coverage metrics obtained using Bowtie2 v.2.5.3 (Langmead and Salzberg 2012) and SAMtools v.1.21 (Danecek et al. 2021), with normalization for graphical representation, considering the removal of redundant reads, using CD-HIT v.4.8.1 (Fu et al. 2012). The nucleotide composition and relative usage content of synonymous codons (RSCU) were estimated using the statistical language R v.4.3.3 (R Core Team 2024), with general nucleotide composition biases calculated using the formulas AT skew = (A% - T%)/(A% + T%) and GC skew = (G% - C%)/(G% + C%) (Perna and Kocher 1995). Additionally, the evaluation of the evolutionary pressure acting on PCGs was performed based on the calculation of the ratio between non synonymous (dN) and synonymous (dS) substitutions (ω = dN/dS), using CodeML (PAML package v.4.9j) (Yang 2007). All graphs illustrating the results obtained were produced using R, along with the libraries ape v.5.7.1 (Paradis et al. 2004), ggplot2 v.3.5.0 (Wickham 2016), pheatmap v.1.0.12 (Kolde 2019) and reshape2 v.1.4.4 (Wickham 2007).

#### Phylogenetic analysis

Phylogenetic analyses were performed using all 13 protein-coding genes (PCGs) from the obtained sequences and from another 189 mtDNAs (RefSeq) from species available in GenBank (NCBI). NCBI reference sequences were used to ensure taxonomic standardization and phylogenetic representativeness of the included taxa. The sequences were aligned using MAFFT v.7.520 (Katoh et al. 2013) and manually inspected using Aliview v.1.28 (Larsson 2014). Subsequently, using IQ-TREE v.1.6.12 (Nguyen et al. 2015), the best nucleotide substitution model was determined according to the Akaike Information Criterion (AIC), followed by phylogenetic signal assessment (Strimmer and Von Haeseler 1997) and phylogeny reconstruction by Maximum Likelihood, with support values defined for 1,000 pseudoreplicates. Finally, the resulting topology was visualized using the ggtree v.3.16.3 library (Yu et al. 2017), with the root defined based on the midpoint of the distances between the taxa.

## Results and discussion

### Morphological identification of nymphs of the species *A. humerale* and *A. geayi*

**Fig. 2:**
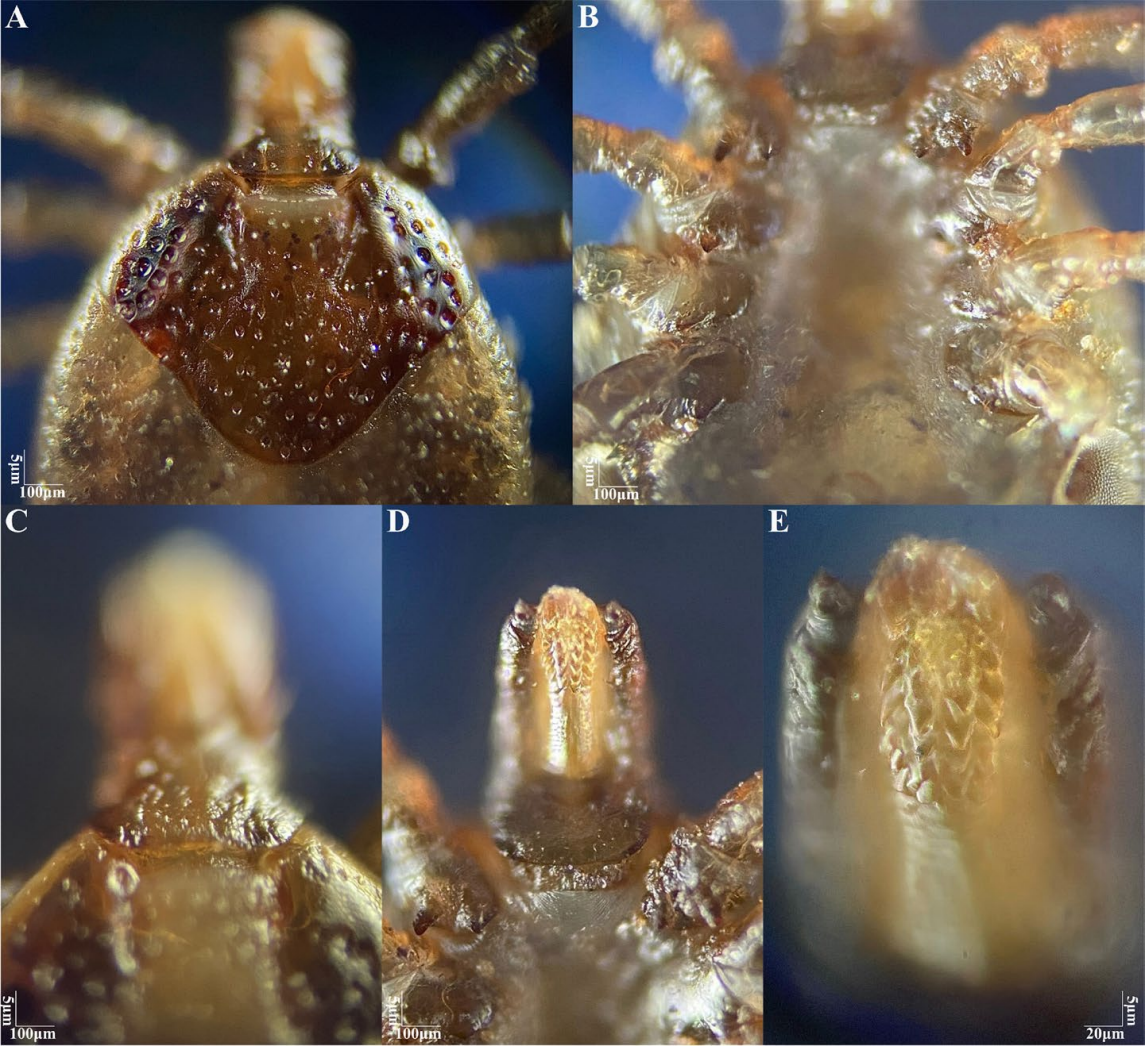
Morphological characteristics of the nymph of the species *A. humerale*. **A**) Dorsal view of the shield with a broadly smooth surface, with large, deep pits evenly distributed, shield relatively wider than long (10x); **B**) Ventral view of the podosoma, coxa I-IV with spurs (10x); **C**) Dorsal region of the base of the capitula in a subtriangular shape (10x); **D**) Ventral region of the base of the capitula with absence of auricles (10x); **E**) Rounded hypostome with dentition arranged in a 2/2 pattern (40x)

The *A. humerale*: Scutum with large, deep pits distributed uniformly, with non-orbited eyes at the mid-length of the scutum; deep cervical grooves reaching the mid-length of the scutum (Fig. 2.A). Coxa I with 2 triangular spurs, the outer one slightly longer; coxae II–III with 2 small spurs, the outer one triangular and longer, the inner one rounded and almost obsolete, especially in coxa III. Coxa IV with a small triangular spur (Fig. 2.B). Base of the capitula in subtriangular shape with absence of cornuae (Fig. 2.C). Base of the capitula with ventrally convex posterior margin, without auricles (Fig. 2.D). Apically rounded hypostome with 2/2 dentition (Fig. 2.E) (Martins et al. 2010).

**Fig. 3:**
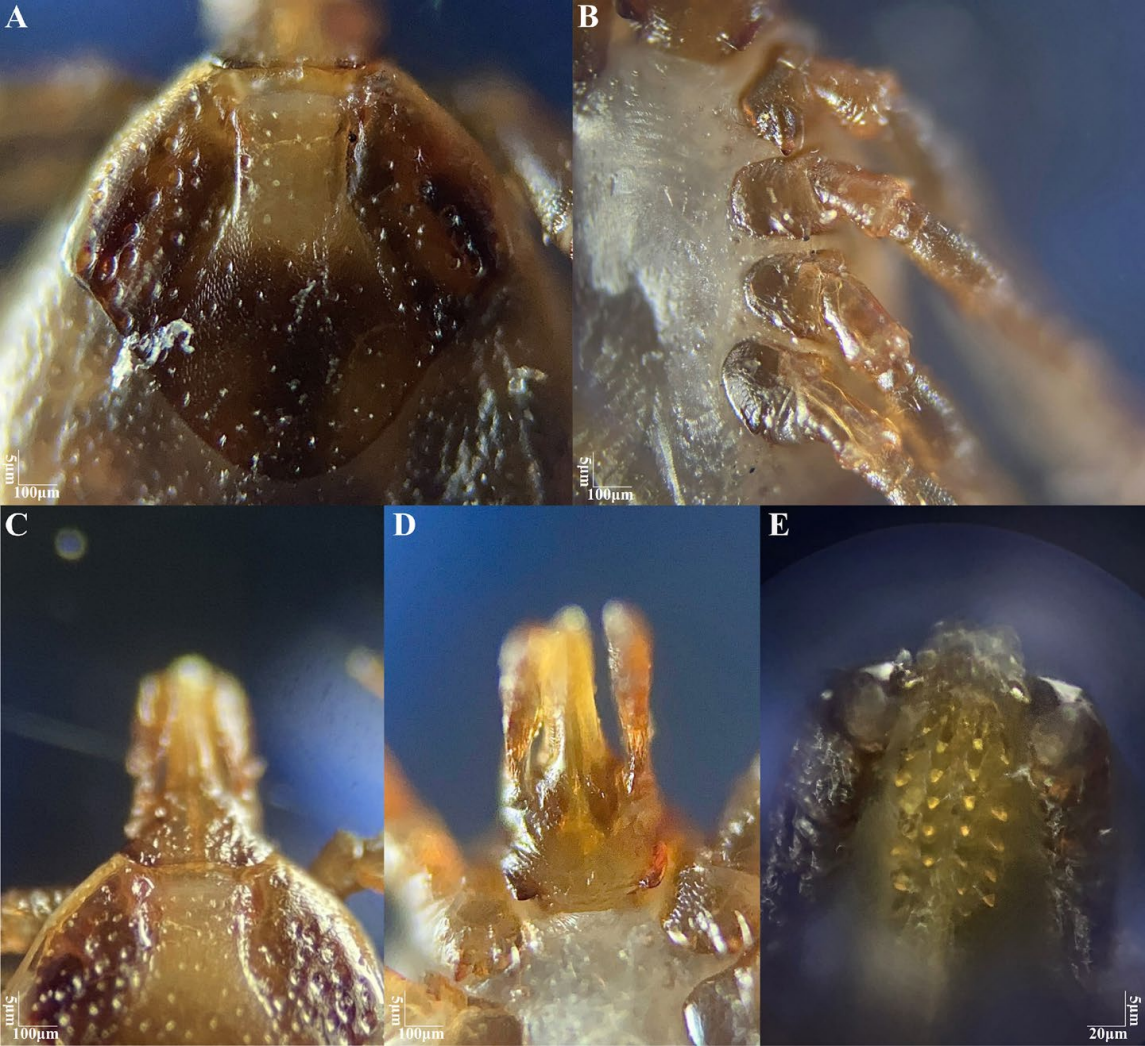
Morphological characteristics of the nymph of the species *A. geayi*. **A**) Dorsal view of the shield with a broadly rough surface, with moderately distributed punctures, larger and deeper in the lateral regions (10x); **B**) Ventral view of coxae I-IV with spurs (10x); **C**) Dorsal region of the base of the capitula in a subtriangular shape, absence of cornuae (10x); **D**) Ventral region of the base of the capitula, auricles as rounded posterolateral projections (10x); **E**) Rounded hypostome with dentition arranged in a 2/2 pattern (40x)

The *A. geayi*: Scutum with extensively wrinkled (rugose) surface; punctations moderately distributed, with larger and deeper punctations on the lateral margins; cervical grooves reaching the middle portion of the scutum, deep anteriorly (Fig. 3.A). Coxa I with 2 short spurs, the outer one wider and longer, the inner one obsolete; coxae II–IV with a small triangular spur (Fig. 3.B). Capitulum base subtriangular in shape, without horns (Fig. 3.C). Capitulum base with ventrally convex posterior margin, auricle with small rounded posterolateral projections (Fig. 3.D). Hypostome rounded apically and dentition distributed in a 2/2 pattern (Fig. 3.E) (Martins et al. 2013).

### Molecular identification of the investigated species

**Fig. 4:**
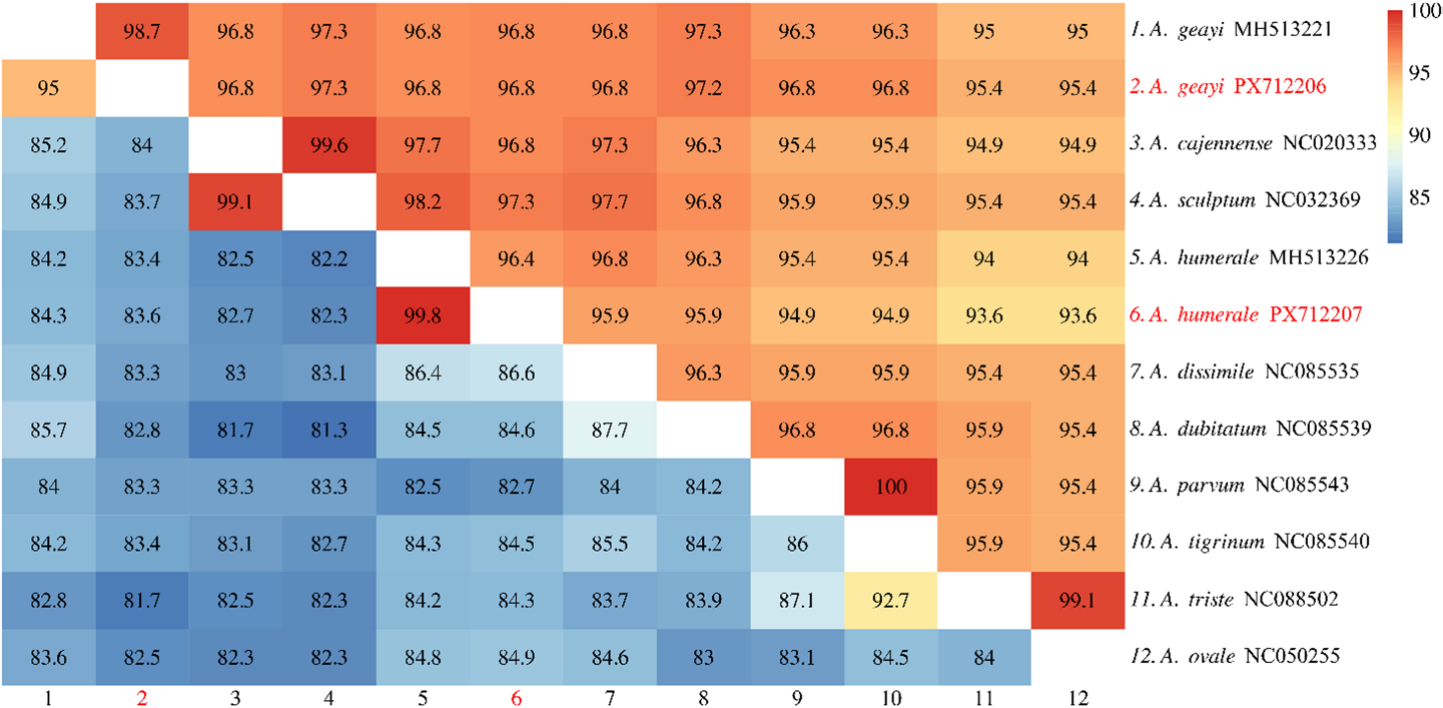
Heat map of nucleotide/amino acid identity among taxa of the genus *Amblyomma*, based on the barcode region of the *COXI* gene. The lower and upper triangles contain the percentages of nucleotide and amino acid identity, respectively. Taxa highlighted in red indicate the sequences obtained

The identification of the obtained sequences was confirmed through a comparative model using the 5’ terminal region of the *COXI* gene (658 bp) with ten other sequences of the genus *Amblyomma* available in the GenBank database (NCBI). *A. geayi* (GenBank PX712206) and *A. humerale* (GenBank PX712207) showed nucleotide identity of 95% and 99.8%, respectively (Fig. 4).

### Organization of mitochondrial genomes

**Fig. 5:**
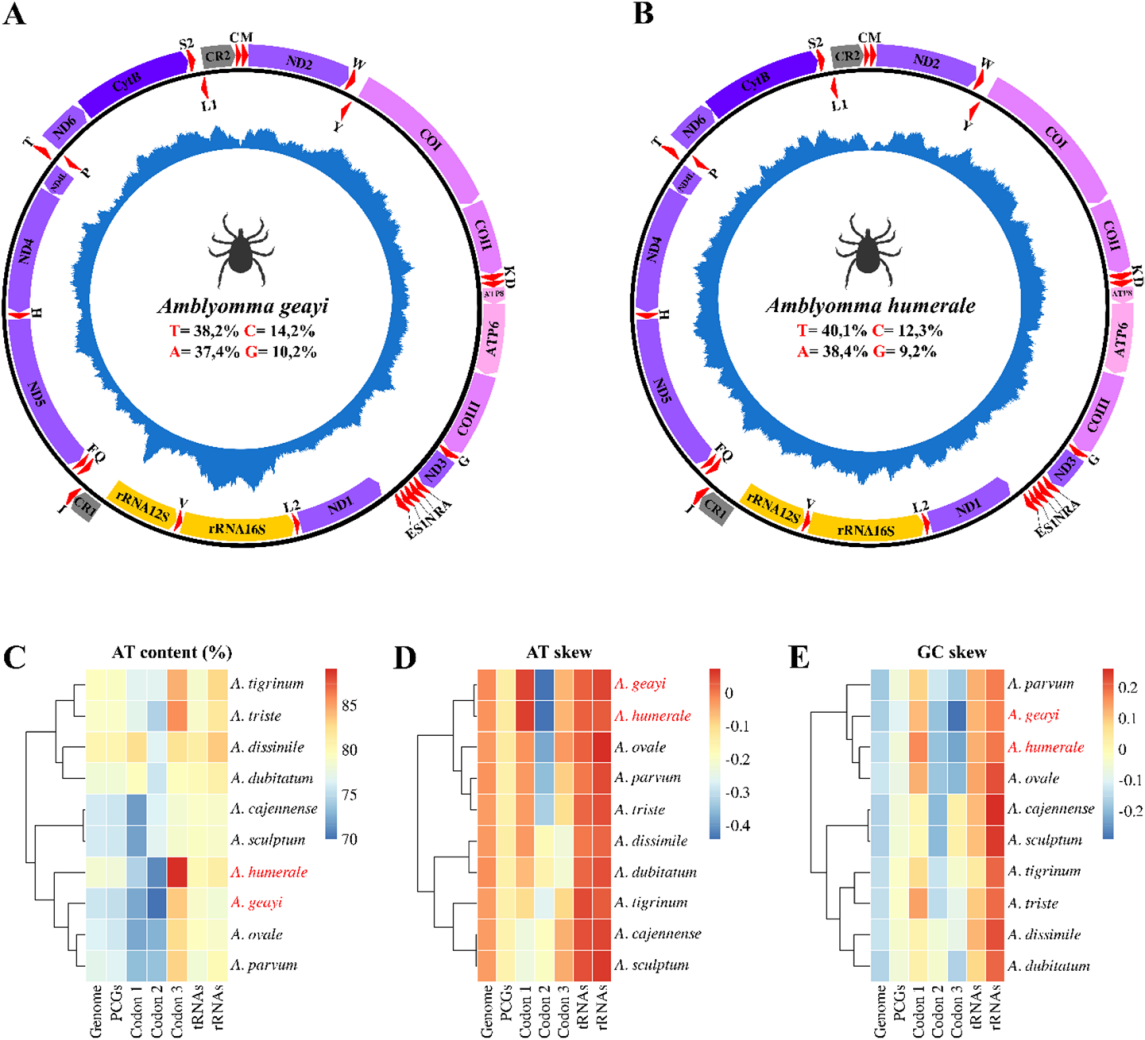
Circular structural representation of the mitochondrial genomes of the investigated species and nucleotide content. The internal values indicate the content of the nucleotide bases (**A-B**). The blue inner graph indicates the distribution of genomic coverage by region. The purple, red, yellow, and gray blocks indicate PCGs, tRNAs, rRNAs, and CR1 and CR2 regions. Genes outside and inside the circle have forward and reverse transcription directions, respectively. (**C**) AT% composition, (**D**) AT-skew, and (**E**) GC-skew of 10 mtDNAs from previously characterized ticks, including *A. geayi* and *A. humerale* (highlighted in red). Hierarchical species clusters (y-axis) are established based on the quantity of each metric per evaluated region (x-axis). The scales, particularly AT-skew and GC-skew, consider the actual (non normalized) values obtained in each analysis

The mtDNAs of the species *A. geayi* and *A. humerale* presented a total size of 14,694 bp and 14,795 bp, respectively. The investigated species presented a circular molecule structured in 37 functional subunits: 13 PCGs, 22 tRNAs and two rRNAs, with an absence of gene inversions/translocations along the coding chains, including two adenine- and thymine-rich replication control regions (CR1 and CR2) and two regions called “Tick-box” (Fig. 5.A-B). This conformation is observed in several species of metastriate ticks from the family Ixodidae such as *Rhipicephalus sanguineus* (Liu et al. 2013), *Amblyomma americanum* (Williams-Newkirk et al. 2015), *Amblyomma geoemydae* (Chang et al. 2019), *Hyalomma marginatum* (Ciloglu et al. 2021), *Rhipicephalus microplus* (Deng et al. 2024), *Rhipicephalus linnaei* (Silva e Silva et al. 2026), and *Haemaphysalis tibetensis* (Tang et al. 2024).

The obtained sequences showed an AT content of 75.6% and 78.5% when evaluating the total genome, including the A + T regions, of the species *A. geayi* and *A. humerale*, respectively (Fig. 5.C) (Online Resource 2 and 3). Similar results have been reported in other species of the genus *Amblyomma* such as *Amblyomma ovale* (Uribe et al. 2020), *Amblyomma cajennense* (Burger et al. 2013), *Amblyomma maculatum* (Brenner and Raghavan 2021) and *Amblyomma sculptum* (Lima et al. 2017).

The asymmetry of nucleotide composition in a mtDNA strand is generally described and quantified by the AT-skew and GC-skew indices (Wei et al. 2010; Zhuang et al. 2013). The total genome of the species *A. geayi* is observed to be almost symmetrical, but there is still a slightly negative AT-Skew slope (−0.0101), indicating that the A and T content are relatively close, while GC Skew is predominantly negative. *A. humerale* presents similar results with a relatively negative AT-Skew (−0.0218) and a largely negative GC-Skew (−0.1454). These data indicate slightly higher proportions of Thymine relative to Adenine and a pronounced proportion of Cytosine relative to Guanine. Apparently, the AT slope of the obtained species deviated from the general characteristics of mitochondrial genomes of metazoans (Nascimento et al. 2021; Silva e Silva et al. 2022; Silva et al. 2023; Wang et al. 2024; Tuangpermsub et al. 2025). These asymmetry characteristics are a peculiarity of hard ticks, which in their great majority have negative AT deviations, with the exception of the species *Ixodes tasmani* (0.001), *Ixodes uriae* (0.007) and *Ixodes hexagonus* (0.033), which have positive AT Skew, while soft ticks have predominantly positive AT-Skew (Wang et al. 2019, 2024; Cao et al. 2023; Tuangpermsub et al. 2025).

The vast majority of the 22 tRNA subunits identified in the obtained sequences presented the typical “clover” conformation (Online Resource 4 and 5). However, in *A. geayi*, tRNACys and tRNASer1 showed absence of the DHU arm; tRNAAla exhibited shortening of the TΨC arm, being replaced by a TΨC loop; and tRNAPhe showed absence of this same arm. In *A. humerale*, most subunits also maintained the “clover” conformation, except for tRNACys and tRNASer1, in which the DHU arm was absent, as observed in *Amblyomma triste* (Ossa-López et al. 2024). The total length considering the 22 concatenated tRNAs of the species *A. geayi* was 1364 bp, with an AT content of 78.8%, while for *A. humerale* the 22 concatenated tRNAs totaled 1381 bp, with an AT content of 80.4%. Furthermore, G-U and U-U mismatch regions were observed, which are similar to those of other invertebrates (Silva e Silva et al. 2022; Silva et al. 2023, 2024). These incompatibilities may be associated with evolutionary mutations and possibly do not compromise the function of tRNA genes, since they can be corrected during subsequent maturation or tRNA editing processes (Watanabe et al. 1994).

Twenty intergenic regions were identified, ranging from 1 to 31 bp, with the largest located between *COXI* and *COXII*, for both species obtained. Similarly, four and six overlapping gene regions were identified for *A. humerale* and *A. geayi*, respectively. These regions had an extent of less than 4 base pairs, indicating short and punctual overlaps in the mitochondrial genome.

#### Description of protein-coding regions

The obtained sequences presented nine PCGs located in the forward transcriptional direction (ND2, COI, COII, ATP8, ATP6, COIII, ND3, ND6, and CytB) and four in the reverse direction (ND4L, ND4, ND5, and ND1) (Fig. 05A-B). The length of the PCGs of *A. geayi* ranged from 156 bp (ATP8) to 1650 bp (ND5), with AT content of 78.8% and 77.5%, respectively. It was observed that the species *A. humerale* presented similar results, with 156 bp (ATP8) to 1653 bp (ND5), and AT content of 81.4% and 80.6%, respectively. Results like these have been described in several tick genera (Lima et al. 2017; Ciloglu et al. 2021; Deng et al. 2024; Tang et al. 2024; Silva e Silva et al. 2026).

For the species *A. geayi*, all PCGs presented ATN-pattern start codons, and the complete TAA-type stop codon terminated the coding chain of all PCGs, except for ND6 and CytB, which presented a TAG-type stop codon. *A. humerale* presented similar results with ATN-pattern start codons; however, all PCGs terminated the coding chain with a TAA-type stop codon exclusively (Online Resource 6). Although the sequences obtained in the present study present complete stop codons, some mtDNA genes of ticks of the genus *Amblyomma*, such as *Amblyomma elaphense*, *Amblyomma fimbriatum*, *Amblyomma sphenodonti* and *Amblyomma sparsum*, have been described with incomplete terminations (T* and TA*) (Burger et al. 2012; Cotes-Perdomo et al. 2023), this characteristic has also been reported in other tick species such as *Rh. sanguineus*, *Hyalomma rufipes*, *Hyalomma asiaticum*, *Haemaphysalis verticalis*, *Haemaphysalis flava* and *Haemaphysalis longicornis* (Lang et al. 2022; Cao et al. 2023). In these situations, the stop codon is possibly completed by post transcriptional polyadenylation, a process in which the addition of the poly-A tail to the mRNA results in the formation of the complete stop codon (TAA), ensuring the correct termination of translation and the proper synthesis of mitochondrial proteins (Chang and Tong 2012; Bratic et al. 2016). Furthermore, we emphasize that the PCG annotations of the obtained sequences were performed based on the ORFs of each coding gene.

**Fig. 6:**
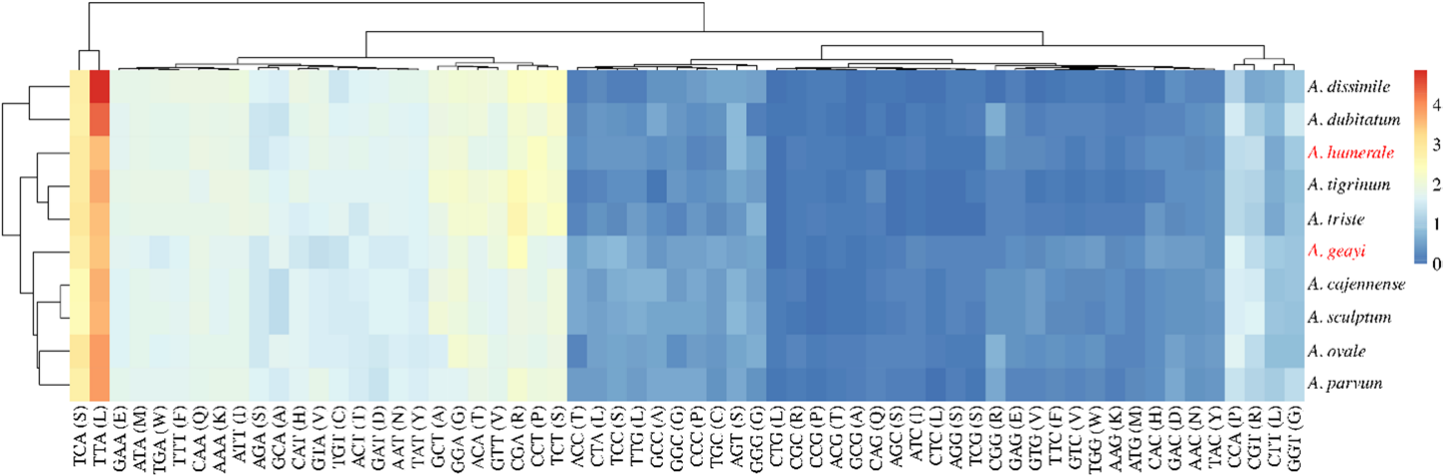
Relative use of synonymous codons of *A. geayi* and *A. humerale* (highlighted in red) compared to other representatives of the genus *Amblyomma*. Hierarchical groupings (clusters) of species (y-axis) are established based on the RSCU of each codon (x-axis), with indications regarding the scale values

In the relative codon usage analysis (RSCU), the species *A. geayi* expressed all 62 amino acid triplets predicted in the mitochondrial genetic code of invertebrates, while the species *A. humerale* expressed 61 triplets, with the CTG (Leucine) codon absent (Online Resource 6). When comparing the RSCU metrics of *A. geayi* and *A. humerale* with other species of the genus *Amblyomma*, it is observed that most codons whose third base is Adenine or Thymine (Uracil) are expressed more frequently (RSCU > 1) (Fig. 6) than those ending in Cytosine and Guanine (RSCU < 1). Similar results have been described in *A. ovale* (Uribe et al. 2020), *Rh. sanguineus* (Cao et al. 2023), *Hyalomma anatolicum* (Che et al. 2025), *Hy. marginatum* (Ciloglu et al. 2021) and *Rh. linnaei* (Silva e Silva et al. 2026), as well as in other arthropod groups (Silva e Silva et al. 2022; Silva et al. 2024).

**Fig. 7:**
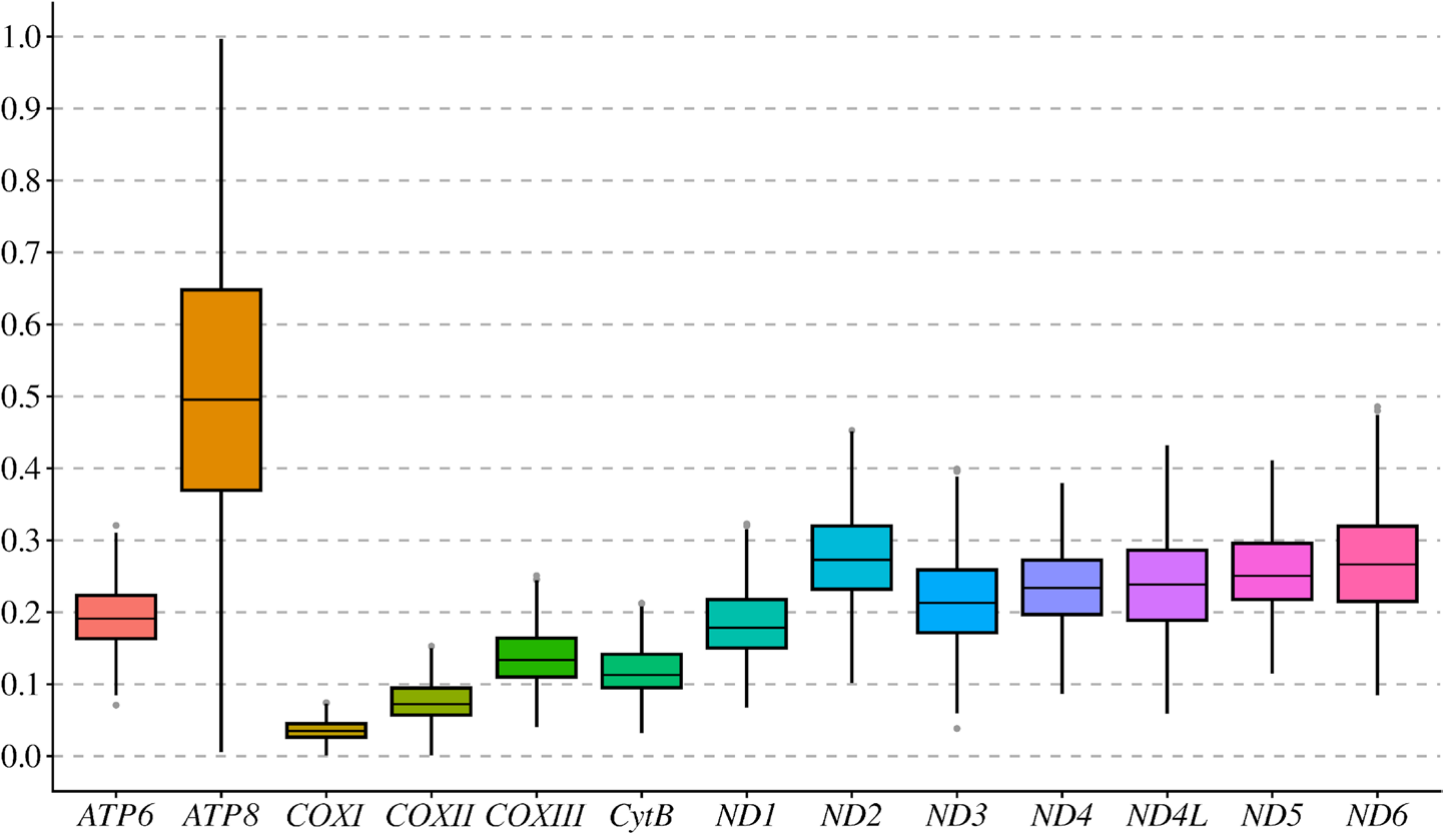
dN/dS ratios calculated from paired analyses of 35 mtDNAs from specimens of the subfamily Amblyomminae. The ω and PCG ratios are arranged along the y and x axes, respectively

The evolutionary pressure acting on the PCG sequences investigated and those of other representatives of the genus *Amblyomma* was also evaluated based on the estimation of the ratios of non-synonymous to synonymous substitutions (dN/dS). The results obtained indicate that the different coding regions are evolving globally under the influence of negative (or purifying) pressure, with ratios ranging from 0.0010 ± 0.0744 in *COXI* and 0.0055 ± 0.9972 in *ATP8*. Furthermore, it was observed that the PCGs of *A. humerale* and *A. geayi*, along with other representatives of the genus *Amblyomma*, are evolving globally under strong negative selection pressure (dN/dS < 0.1), resulting in high rates of synonymous substitutions evaluated (Zhang and Yu 2006). Purifying selection was particularly strong (dN/dS < 0.1) in the PCGs of complexes III (*CytB*) and IV (*COX*) and in the *ATP6* subunit belonging to complex V. In turn, *ATP8* and the genes belonging to complex I (*ND*) showed higher dN/dS ratios (0.1 < dN/dS < 0.5), indicating the presence of less conservative evolutionary constraints in these regions. However, it was also observed that the rates of synonymous substitutions (dS) in the PCGs were still significantly higher compared to the occurrence of non synonymous substitutions (dN) (Fig. 7). It is relevant to highlight that the rates of mutation accumulation vary among the different protein-coding gene complexes (PCGs), reflecting the specific role that each plays in metabolic processes and in the oxidative phosphorylation chain reactions of cellular respiration (Wolstenholme 1992).

#### Description of *12 S rRNA* and *16 S rRNA*

The *16 S rRNA* gene, located between tRNA-L2 and tRNA-V, and the *12 S rRNA* gene, located between tRNA-V and CR1, situated on the reverse strand (Fig. 5.**A-B**) of the obtained sequences, presented lengths of 1,216 bp and 698 bp in *A. humerale* and 1187 bp and 756 bp in *A. geayi*, respectively. For *A. humerale*, the lengths of the *16 S rRNA* and *12 S rRNA* genes were 1,216 bp and 698 bp, respectively, while for *A. geayi*, the lengths were 1,187 bp and 756 bp, respectively. The 16 S gene showed an AT rate of 79.5% and 82.2%, respectively for the species *A. humerale* and *A. geayi*, while the 12 S gene resulted in 79.7% and 76.6% AT. Similar results have been reported in other species of the genus *Amblyomma* (Burger et al. 2013; Lima et al. 2017; Duan et al. 2019; Uribe et al. 2020; Cotes-Perdomo et al. 2023; Ossa-López et al. 2024). These results have also been observed in genera of the family Argasidae, such as *Ornithodoros* (Mans et al. 2012, 2025) and *Carios* (Mans et al. 2021), as well as in the family Nuttalliellidae, represented by *Nuttaliella namaqua* (Mans et al. 2012).

Frequently, different molecular markers are used for the taxonomic identification of metazoans (Cruickshank 2002; Tyagi et al. 2019; Ferreira et al. 2024; Reichl et al. 2024). In ticks, molecular taxonomic studies have focused especially on the *COXI*, *CytB*, *12 S rRNA*, and *16 S rRNA* genes of the mitochondrial genome (Abouelhassan et al. 2019; Koroiva et al. 2022). Studies highlight that the *12 S rRNA* and *16 S rRNA* genes constitute good alternatives for specimen identification, especially when reference markers, such as the *COXI* gene, do not provide reliable results (Lv et al. 2014; Abouelhassan et al. 2019; Jomli et al. 2025).

#### Description of non-coding regions (NCRs)

The investigated sequences presented two non-coding regions located in the forward transcriptional direction. The first non-coding region (CR1) is positioned between *12 S rRNA* and tRNA-Ile (I), and the second non-coding region (CR2) is positioned between tRNA-Leu1 (L1) and tRNA-Cys (C), for both sequences obtained (Fig. 5.**A-B**). It was observed that the length of the NCRs varied among the species studied. The species *A. humerale* presented 309 bp and 302 bp for CR1 and CR2, while *A. geayi* exhibited 245 bp and 312 bp for CR1 and CR2, respectively. Although this pattern of NCR positioning is frequently reported in species of the genus *Amblyomma* (Duan et al. 2019; Cotes-Perdomo et al. 2023).

Tick NCRs have been extensively studied, as their number, size, and location vary among different species (Kelava et al. 2021; Jiang et al. 2022; Lu et al. 2022; Mans et al. 2025). Furthermore, translocations involving NCRs are frequently reported, suggesting the occurrence of mitochondrial rearrangements (Wang et al. 2019). These variable characteristics are recorded in several specimens of the families Argasidae, Nuttalliellidae, and Ixodidae (Kelava et al. 2021). Two non-coding regions called “Tick-boxes” were identified; similar results have been reported in some Metastriata (Montagna et al. 2012; Brenner and Raghavan 2021; Chavatte and Octavia 2021). These are small regions of 21 bp for both sequences obtained. It is believed that these regions direct the formation of the 3’ end of polyadenylated *ND1* transcripts in Ixodidae, and possibly act as a maturation signal for the cleavage of a large precursor transcript or a transcription termination signal (Montagna et al. 2012).

### Phylogenetic Analysis

The reconstruction of the phylogeny, using the Maximum Likelihood method, resulted in topologies with high internal support values; the subfamily Amblyomminae formed a clade with the subfamilies Rhipicephalinae and Hyalomminae, and was externally anchored by the subclade Haemaphysalinae + Bothriocrotoninae + *Cryptocroton papuanum* (Online Resource 7). Analyses based on the 13 protein-coding regions demonstrate that the genus *Dermacentor* formed a monophyletic clade with high support values (bootstrap < 99%), with the species *Rhipicentor nuttalli* as its immediate sister taxon, in which the genera *Hyalomma* and *Rhipicephalus* form a clade that, topologically, is sister to the set (*Dermacentor* + *Rhipicentor nuttalli*). This conformation has already been reported in previous phylogenetic studies (Wang et al. 2019; Chavatte and Octavia 2021). Although studies mention the genus *Dermacentor* as belonging to the subfamily Rhipicephalinae (Parola and Raoult 2001; Barker and Murrell 2004).

**Fig. 8:**
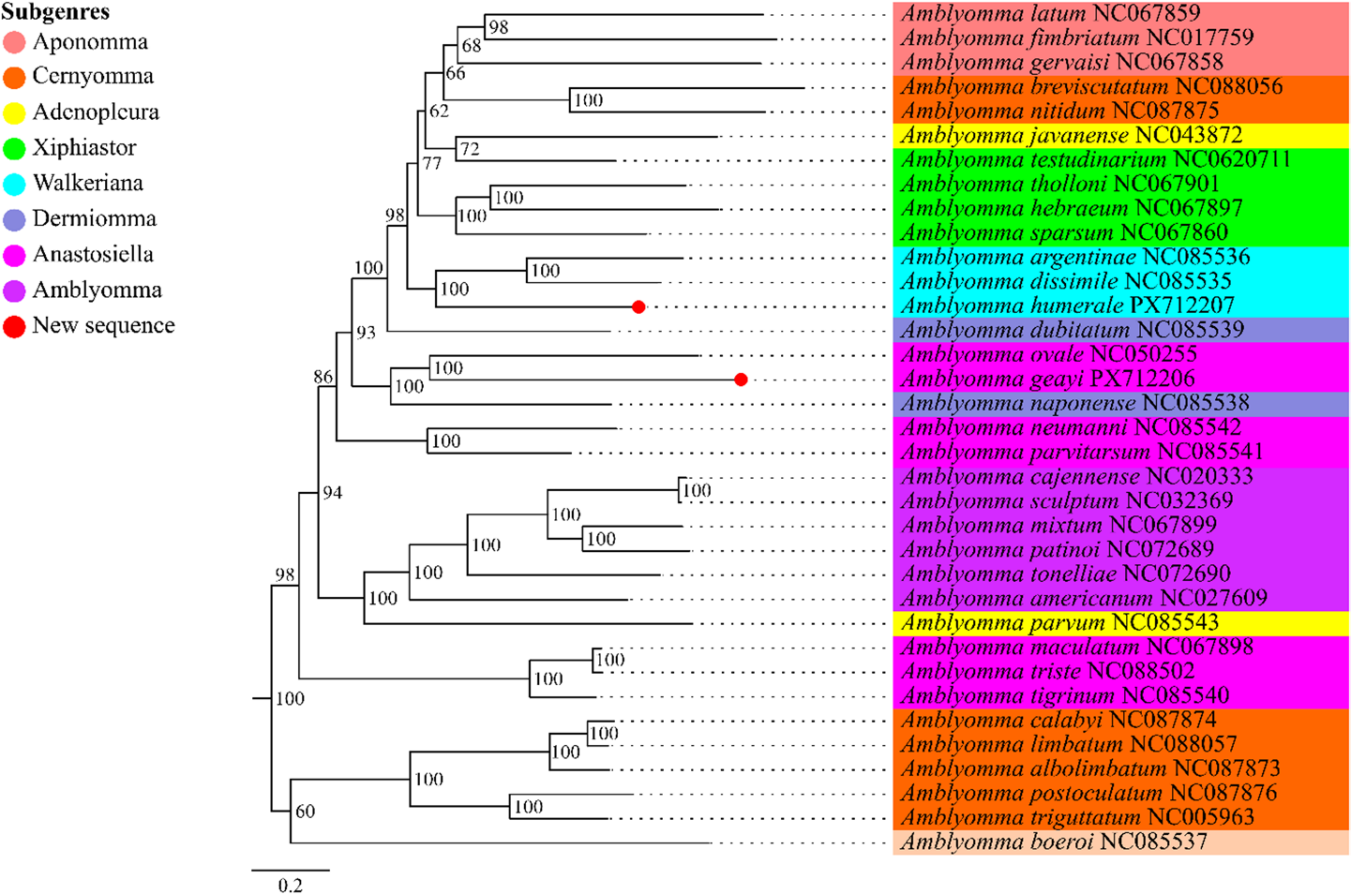
Phylogenetic reconstruction of the subfamily Amblyomminae based on the 13 PCGs of *A. humerale* and *A. geayi* (highlighted in red) and other taxa available in the GenBank database (NCBI). Initial support (BS) values are shown at each node

The genus *Amblyomma* (35 taxa) was organized into a well-defined clade with high support values, including the investigated sequences, supporting the monophyly of the group (Fig. 8). It is observed that the sequence of the species *A. humerale*, obtained in the present study, formed a subclade with the species *Amblyomma dissimile* and *Amblyomma argentinae* with maximum support values (100%), while the sequence of the species *A. geayi* formed a phylogenetic subclade with the species *A. ovale* (100%).

Considering the 13 PCGs, it is noted that the species *A. geayi* grouped in the subgenus *Anastosiella*, along with the species *A. ovale*. In phylogenetic reconstructions, which used mtDNA from different localities, it was observed that different sequences of the species *A. ovale* formed a solid group with high support values (Ossa-López et al. 2024). Previous studies, which used different molecular markers of mtDNA, suggest that *A. geayi* is phylogenetically closer to the species *Amblyomma longirostre* and *Amblyomma parkeri*, with high phylogenetic signal values (Labruna et al. 2009).

The sequence of the species *A. humerale* grouped with representatives of the subgenus *Walkeriana* (*A. argentinae* and *A. dissimile*), showing high bootstrap values (100%). This conformation of the *Walkeriana* subgenus has also been described in studies that used partial sequences of other genes (Uribe et al. 2024). The results of the present study corroborate previous studies that recovered several species of the genus *Amblyomma*, including *A. humerela*, from molecular markers of the mitochondrial genome (Robayo-Sánchez et al. 2020; Santodomingo et al. 2021). In addition, the present study identified translocations of representatives classified in the *Cernyomma* subgenus, in which the species *Amblyomma breviscutatum* and *Amblyomma nitidum* formed a sister subclade of the *Aponomma* subgenus, while the other species grouped together forming a sister clade with the species *Amblyomma boeroi*. Although studies mention phylogenetic relationships between *A. boeroi*, *Amblyomma parvitarsum*, and *Amblyomma neumanni* (Beati and Klompen 2019; Santodomingo et al. 2021), we emphasize that the use of different sets of molecular markers can alter the supports and topologies (Murrell et al. 2001); these characteristics have been observed in several studies (Uribe et al. 2024).

In recent years, mitochondrial genomes have demonstrated significant advantages and have been widely used in taxonomic and phylogenetic studies (Silva e Silva et al. 2022; Cao et al. 2023; Almazán et al. 2024). Currently, the NCBI database contains several complete mitochondrial genomes of ticks, which have been widely used in phylogenetic and comparative studies. However, challenges persist in systematic investigations based on mitogenomes, mainly due to the still incomplete taxonomic coverage, since several species remain without available mitochondrial data. The absence of complete mitochondrial data for ticks limits the resolution and robustness of cladistic analyses, hindering the precise definition of evolutionary relationships within the order Ixodida (Wang et al. 2019; Gao et al. 2022). This information gap can lead to the formation of artificial clades or the uncertain placement of certain taxa in phylogenetic trees (Semenchenko et al. 2025). In this context, the incorporation of complete mitochondrial genomes represents a fundamental approach to increase the informative power of phylogenetic analyses, since it expands the number of comparable characters, reduces biases associated with isolated markers, and improves the statistical support of the recovered nodes (Duchêne et al. 2011; Kneubehl et al. 2022). Thus, the present study contributes to reducing these uncertainties by employing complete mitochondrial sequences, providing a more robust basis for inferring evolutionary relationships between the analyzed sequences.

## Conclusion

This is the first study to characterize the complete mitochondrial genome of the tick species *A. humerale* and *A. geayi*. The mitogenomes obtained showed a structural organization typical of metastriated ticks, composed of 13 PCG, 22 tRNA genes, two rRNA genes, in addition to two conserved regions called “tick boxes” and two adenine-thymine-rich control regions (A + T). In the phylogenetic context, the complete mitochondrial sequences obtained in this study grouped into a monophyletic clade along with previously described representatives of the genus *Amblyomma*, corroborating the taxonomic position of these species within the order Ixodida. The recovery of this monophyletic grouping reinforces the usefulness of complete mitochondrial genomes as robust markers for evolutionary and systematic inferences in ticks. Furthermore, the sequences generated here provide relevant information for advancing knowledge about the phylogenetic relationships among different representatives of the order Ixodida. The use of complete mitochondrial genomes substantially expands the number of comparable characters in phylogenetic analyses, reducing limitations associated with the use of isolated mitochondrial markers, such as fragments of specific genes. This integrative approach tends to minimize phylogenetic biases, increase the statistical support of the recovered nodes, and produce more stable, resolved, and biologically interpretable phylogenetic topologies. Consequently, the data generated in this study represent an important genomic resource for future investigations in systematics, molecular evolution, and biogeography of Neotropical ticks, in addition to contributing to the improvement of genetic databases used in comparative studies within the order Ixodida.

## Supplementary Information

Below is the link to the electronic supplementary material.

Supplementary Material 1

Supplementary Material 2

Supplementary Material 3

Supplementary Material 4

Supplementary Material 5

Supplementary Material 6

Supplementary Material 7

Supplementary Material 8

Supplementary Material 9

## Data Availability

All data generated during this study are available as tables and Figures included in this published article and its Supplementary Information files. The GenBank database accession numbers for the mitochondrial genomes sequenced in this study are PX712206 (Amblyomma geayi) and PX712207 (Amblyomma humerale). SRA data: PRJNA1468630.
