# Supplementary material for: First description of the complete mitochondrial genomes of the species Amblyomma humerale and Amblyomma geayi (Acari: Ixodidae), Amazon, Pará, Brazil
Source: Exp Appl Acarol. 2026 Jun 24;97(2):8. doi: 10.1007/s10493-026-01151-w (PMC13294308; doi:10.1007/s10493-026-01151-w)
Supplement: Supplementary file 7 — Supplementary Material 7 [file 10493_2026_1151_MOESM7_ESM.pdf]

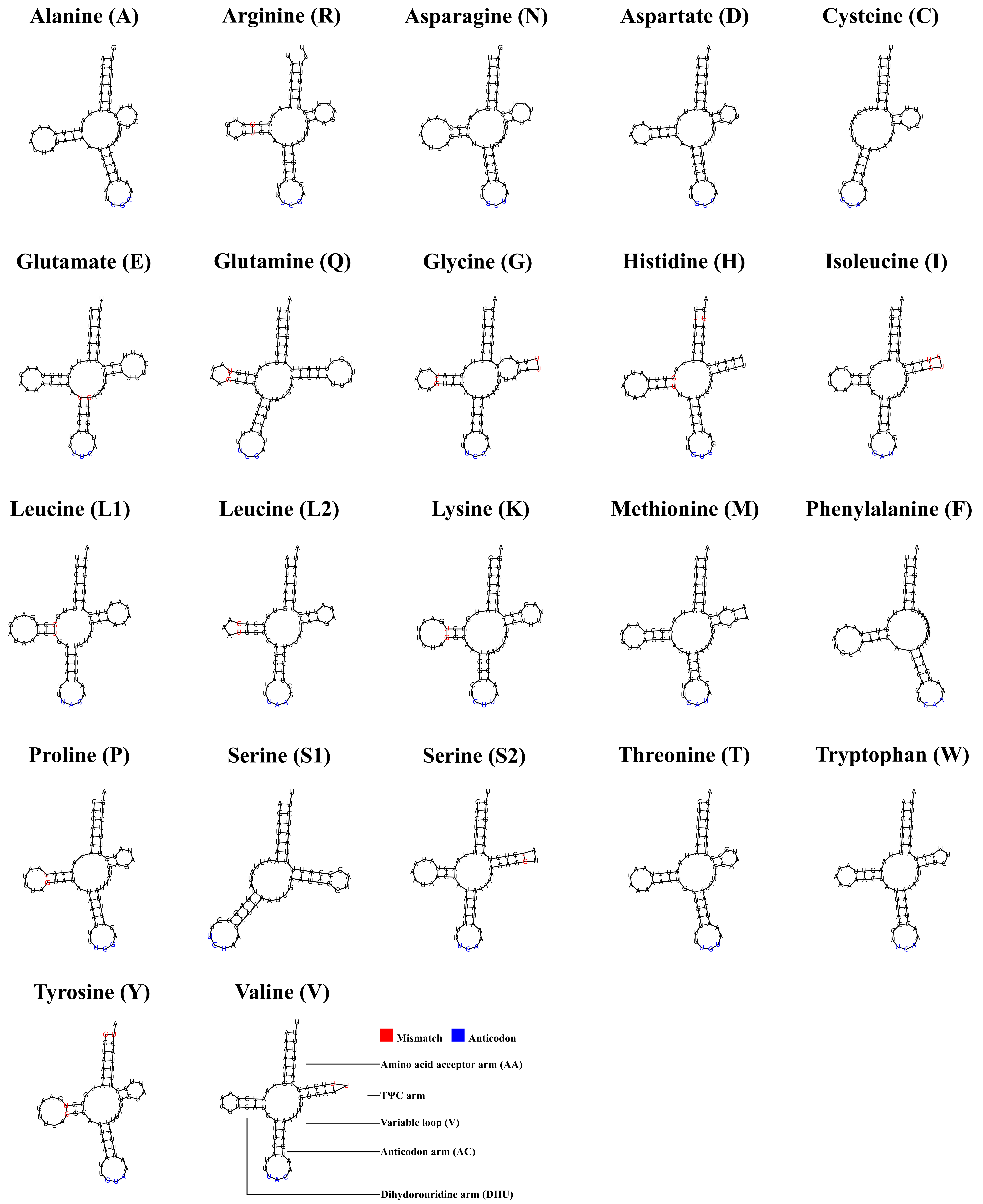

Supplementary Figure S1. Secondary sctructures of tRNAs of *Amblyomma geayi* (GenBank PX712206)
