# Supplementary figures and images for: First description of the complete mitochondrial genomes of the species Amblyomma humerale and Amblyomma geayi (Acari: Ixodidae), Amazon, Pará, Brazil
Source: Exp Appl Acarol. 2026 Jun 24;97(2):8. doi: 10.1007/s10493-026-01151-w (PMC13294308; doi:10.1007/s10493-026-01151-w)

**Supplementary Figure S1.** Secondary sctructures of tRNAs of *Amblyomma humerale* (GenBank PX712207)

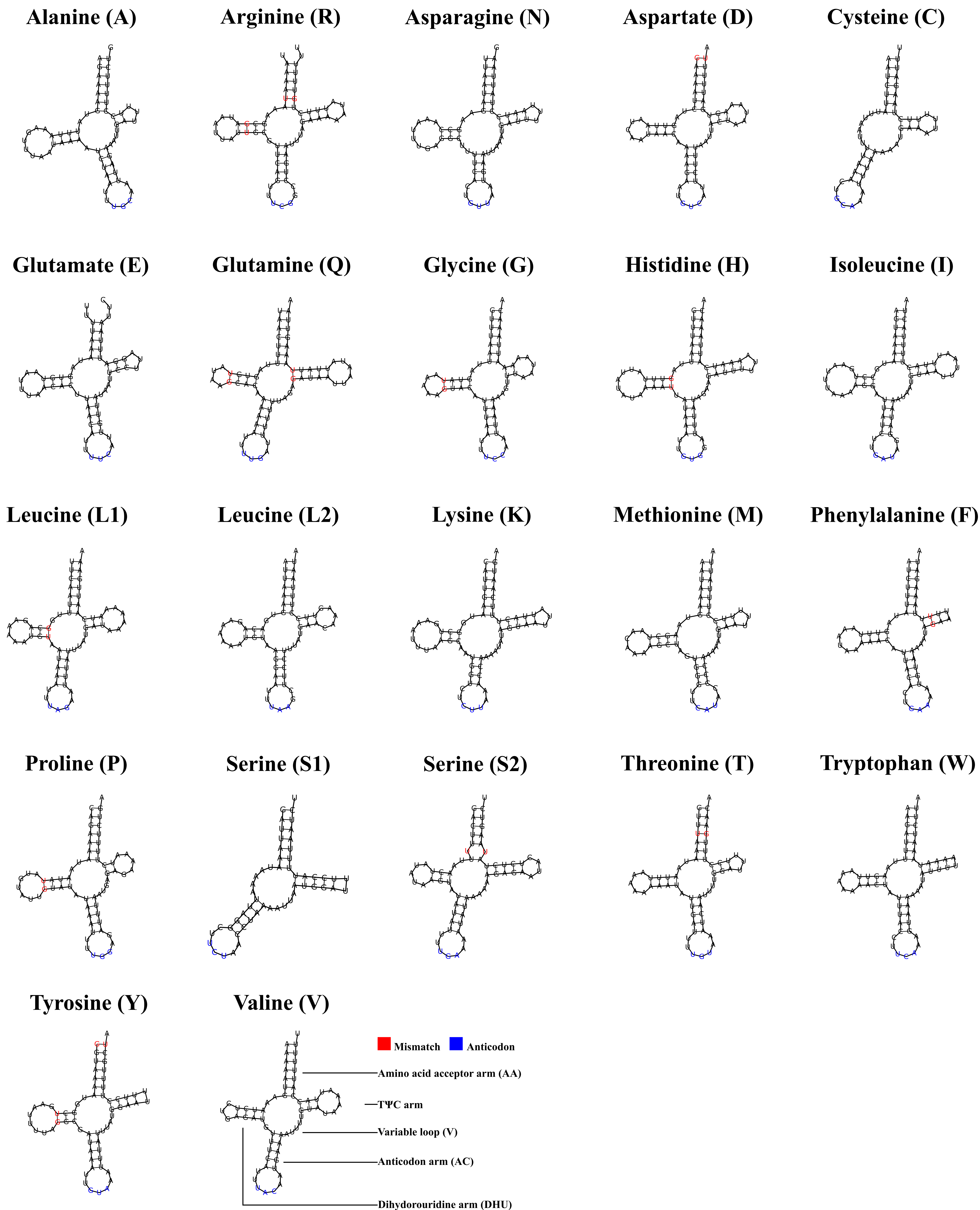

Supplement: Supplementary file 8 — Supplementary Material 8 [file 10493_2026_1151_MOESM8_ESM.pdf]
