# Supplementary material for: First description of the complete mitochondrial genomes of the species Amblyomma humerale and Amblyomma geayi (Acari: Ixodidae), Amazon, Pará, Brazil
Source: Exp Appl Acarol. 2026 Jun 24;97(2):8. doi: 10.1007/s10493-026-01151-w (PMC13294308; doi:10.1007/s10493-026-01151-w)
Supplement: Supplementary file 9 — Supplementary Material 9 [file 10493_2026_1151_MOESM9_ESM.pdf]

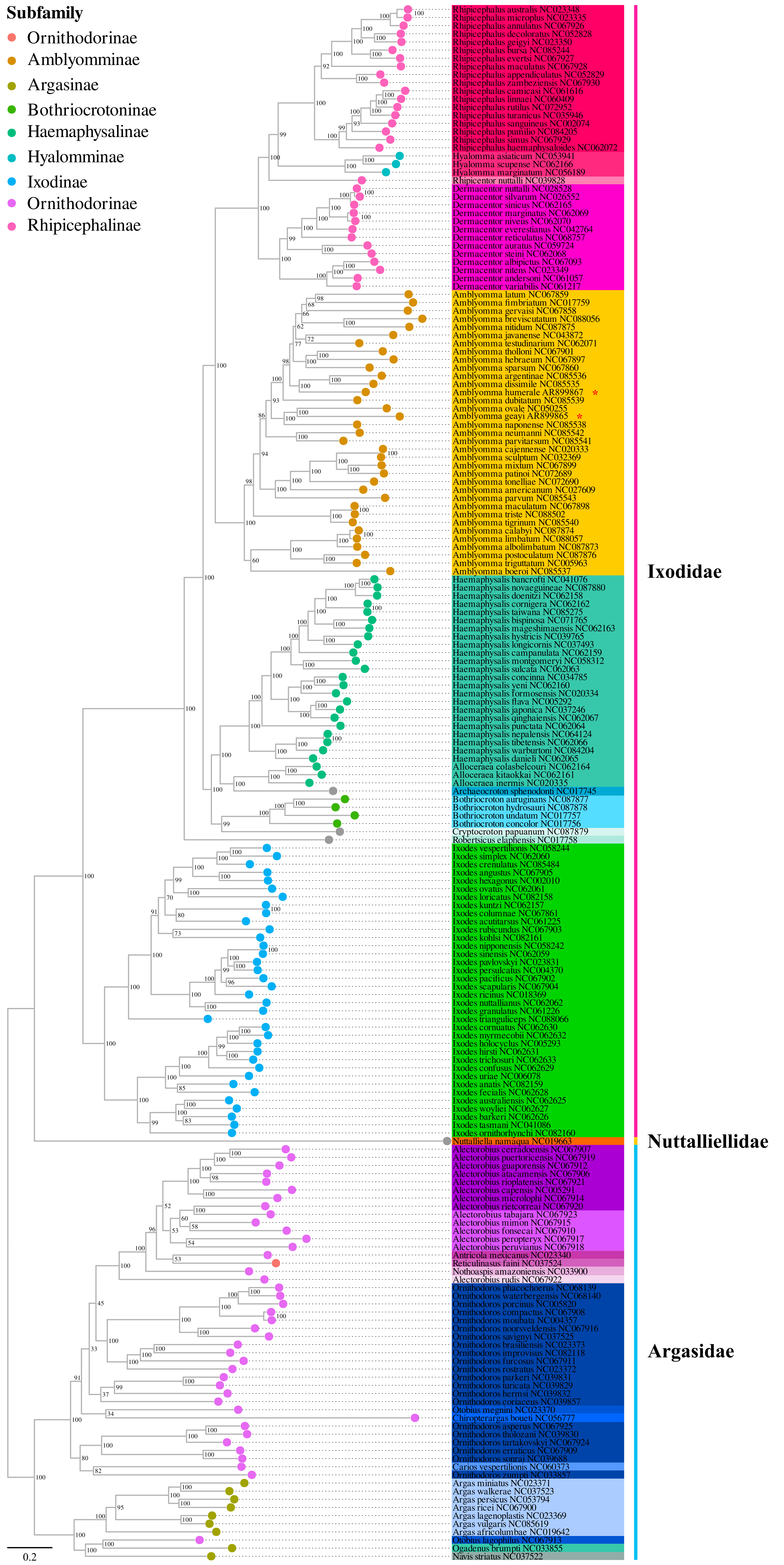
Subfamily
Ornithodorinae
Amblyomminae
Argasinae
Bothriocrotoninae
Haemaphysalinae
Hyalomminae
Ixodinae
Ornithodorinae
Rhipicephalinae
Rhipicephalus australis NC023348
Rhipicephalus microplus NC023335
Rhipicephalus annulatus NC067926
Rhipicephalus decoloratus NC052828
Rhipicephalus geigyi NC023350
Rhipicephalus bursa NC085244
Rhipicephalus evertsi NC067927
Rhipicephalus maculatus NC067928
Rhipicephalus appendiculatus NC052829
Rhipicephalus zambeziensis NC067930
Rhipicephalus camicasi NC061616
Rhipicephalus linnaei NC060409
Rhipicephalus rutilus NC072952
Rhipicephalus turanicus NC035946
Rhipicephalus sanguineus NC002074
Rhipicephalus pumilio NC084205
Rhipicephalus simus NC067929
Rhipicephalus haemaphysaloides NC062072
Hyalomma asiaticum NC053941
Hyalomma scupense NC062166
Hyalomma marginatum NC056189
Rhipicentor nuttalli NC039828
Dermacentor nuttalli NC028528
Dermacentor silvarum NC026552
Dermacentor sinicus NC062165
Dermacentor marginatus NC062069
Dermacentor niveus NC062070
Dermacentor everestianus NC042764
Dermacentor reticulatus NC068757
Dermacentor auratus NC059724
Dermacentor steini NC062068
Dermacentor albipictus NC067093
Dermacentor nitens NC023349
Dermacentor andersoni NC061057
Dermacentor variabilis NC061217
Amblyomma latum NC067859
Amblyomma fimbriatum NC017759
Amblyomma gervaisi NC067858
Amblyomma breviscutatum NC088056
Amblyomma nitidum NC087875
Amblyomma javanense NC043872
Amblyomma testudinarium NC062071
Amblyomma tholloni NC067901
Amblyomma hebraeum NC067897
Amblyomma sparsum NC067860
Amblyomma argentinae NC085536
Amblyomma dissimile NC085535
Amblyomma humerale AR899867 *
Amblyomma dubitatum NC085539
Amblyomma ovale NC050255
Amblyomma geayi AR899865 *
Amblyomma naponense NC085538
Amblyomma neumanni NC085542
Amblyomma parvitarsum NC085541
Amblyomma cajennense NC020333
Amblyomma sculptum NC032369
Amblyomma mixtum NC067899
Amblyomma patinoi NC072689
Amblyomma tonelliae NC072690
Amblyomma americanum NC027609
Amblyomma parvum NC085543
Amblyomma maculatum NC067898
Amblyomma triste NC088502
Amblyomma tigrinum NC085540
Amblyomma calabyi NC087874
Amblyomma limbatum NC088057
Amblyomma albolimbatum NC087873
Amblyomma postoculatum NC087876
Amblyomma triguttatum NC005963
Amblyomma boeroi NC085537
Haemaphysalis bancrofti NC041076
Haemaphysalis novaeguineae NC087880
Haemaphysalis doenitzi NC062158
Haemaphysalis cornigera NC062162
Haemaphysalis taiwana NC085275
Haemaphysalis bispinosa NC071765
Haemaphysalis mageshimaensis NC062163
Haemaphysalis hystricis NC039765
Haemaphysalis longicornis NC037493
Haemaphysalis campanulata NC062159
Haemaphysalis montgomeryi NC058312
Haemaphysalis sulcata NC062063
Haemaphysalis concinna NC034785
Haemaphysalis yeni NC062160
Haemaphysalis formosensis NC020334
Haemaphysalis flava NC005292
Haemaphysalis japonica NC037246
Haemaphysalis qinghaiensis NC062067
Haemaphysalis punctata NC062064
Haemaphysalis nepalensis NC064124
Haemaphysalis tibetensis NC062066
Haemaphysalis warburtoni NC084204
Haemaphysalis danieli NC062065
Alloceraea colasbelcouri NC062164
Alloceraea kitaokai NC062161
Alloceraea inermis NC020335
Archaeocroton sphenodonti NC017745
Bothriocroton auruginans NC087877
Bothriocroton hydrosauri NC087878
Bothriocroton undatum NC017757
Bothriocroton concolor NC017756
Cryptocroton papuanum NC087879
Robertsicus elaphensis NC017758
Ixodes vespertilionis NC058244
Ixodes simplex NC062060
Ixodes crenulatus NC085484
Ixodes angustus NC067905
Ixodes hexagonus NC002010
Ixodes ovatus NC062061
Ixodes loricatus NC082158
Ixodes kuntzi NC062157
Ixodes columnae NC067861
Ixodes acutitarsus NC061225
Ixodes rubicundus NC067903
Ixodes kohlsi NC082161
Ixodes nipponensis NC058242
Ixodes sinensis NC062059
Ixodes pavlovskyi NC023831
Ixodes persulcatus NC004370
Ixodes pacificus NC067902
Ixodes scapularis NC067904
Ixodes ricinus NC018369
Ixodes nuttallianus NC062062
Ixodes granulatus NC061226
Ixodes triangulicepes NC088066
Ixodes cornuatus NC062630
Ixodes myrmecobii NC062632
Ixodes holocyclus NC005293
Ixodes hirsti NC062631
Ixodes trichosuri NC062633
Ixodes confusus NC062629
Ixodes uriae NC006078
Ixodes anatis NC082159
Ixodes fecialis NC062628
Ixodes australiensis NC062625
Ixodes woyliei NC062627
Ixodes barkeri NC062626
Ixodes tasmani NC041086
Ixodes ornithorhynchi NC082160
Nuttalliella namaqua NC019663
Alectorobius cerradoensis NC067907
Alectorobius puertoricensis NC067919
Alectorobius guaporensis NC067912
Alectorobius atacamensis NC067906
Alectorobius rioplatensis NC067921
Alectorobius capensis NC005291
Alectorobius microlophi NC067914
Alectorobius rietcorreai NC067920
Alectorobius tabajara NC067923
Alectorobius mimon NC067915
Alectorobius fonsecai NC067910
Alectorobius peropteryx NC067917
Alectorobius peruvianus NC067918
Antricola mexicanus NC023340
Reticulinasus faini NC037524
Nothoaspis amazoniensis NC033900
Alectorobius rudis NC067922
Ornithodoros phacochoerus NC068139
Ornithodoros waterbergensis NC068140
Ornithodoros porcinus NC005820
Ornithodoros compactus NC067908
Ornithodoros moubata NC004357
Ornithodoros noorsveldensis NC067916
Ornithodoros savignyi NC037525
Ornithodoros brasiliensis NC023373
Ornithodoros improvisus NC082118
Ornithodoros furcosus NC067911
Ornithodoros rostratus NC023372
Ornithodoros parkeri NC039831
Ornithodoros turicata NC039829
Ornithodoros hermsi NC039832
Ornithodoros coriaceus NC039857
Otobius megnini NC023370
Chiropterargas boueti NC056777
Ornithodoros asperus NC067925
Ornithodoros tholozani NC039830
Ornithodoros tartakovskyi NC067924
Ornithodoros erraticus NC067909
Ornithodoros sonrai NC039688
Carios vespertilionis NC060373
Ornithodoros zumpti NC033857
Argas miniatus NC023371
Argas walkerae NC037523
Argas persicus NC053794
Argas ricei NC067900
Argas lagenoplastis NC023369
Argas vulgaris NC085619
Argas africolumbae NC019642
Otobius lagophilus NC067913
Ogadenus brumpti NC033855
Navis striatus NC037522
Ixodidae
Nuttalliellidae
Argasidae
0.2
